# Breaking Down Barriers to Detection and Care in Early-Age-Onset Colorectal Cancer in Canada

**DOI:** 10.3390/curroncol30110680

**Published:** 2023-10-24

**Authors:** Michael J. Raphael, Petra Wildgoose, Filomena Servidio-Italiano, Mary A. De Vera, Darren Brenner, Monika Slovinec D’Angelo, Robin McGee, Scott Berry, Clarence Wong, Sharlene Gill

**Affiliations:** 1 Sunnybrook Health Sciences Centre, Toronto, ON M4N 3M5, Canada; petra.wildgoose@sunnybrook.ca; 2 Colorectal Cancer Resource & Action Network (CCRAN), Toronto, ON M4W 3E2, Canada; filomena.s@ccran.org (F.S.-I.); monika.s@ccran.org (M.S.D.); 3 Faculty of Pharmaceutical Sciences, University of British Columbia, Vancouver, BC V6T 1Z3, Canada; mdevera@mail.ubc.ca; 4 Department of Community Health Sciences, University of Calgary, Calgary, AB T2N 4N1, Canada; darren.brenner@ucalgary.ca; 5 Independent Researcher, Port Williams, NS, Canada; robin.mcgee@eastlink.ca; 6 Department of Oncology, Carlo Fidani Peel Regional Cancer Centre, Mississauga, ON L5M 7S4, Canada; scott.berry@thp.ca; 7 Division of Gastroenterology, University of Alberta, Edmonton, AB T6G 2X8, Canada; ckw3@ualberta.ca; 8 Division of Medical Oncology, BC Cancer, University of British Columbia, Vancouver, BC V5Z 4E6, Canada; sgill@bccancer.bc.ca

**Keywords:** early-age-onset colorectal cancer, EAOCRC, EAO-CRC, EO-CRC, colorectal cancer

## Abstract

The second Early-Age-Onset Colorectal Cancer Symposium, convened in October 2022, sought solutions to the barriers to early detection and care for colorectal cancer in Canada. This meeting built on a previous symposium, held in 2021 and reported in this journal. Early-age-onset colorectal cancer (EAOCRC) affects increasing numbers of people under the age of 50 in Canada and throughout the developed world. Two main themes emerged from the meeting: the importance of timely detection, and the need for a tailored approach to the care of EAOCRC. Early detection is crucial, especially in light of the later stage at diagnosis and unique tumour characteristics. Symposium participants were strongly in favour of reducing the age of eligibility for screening from 50 to 45, and promoting the development of non-invasive screening techniques such as testing for circulating tumour DNA and biomarkers. Leading approaches to care were described and discussed, which meet the unique treatment needs of younger CRC patients. Multidisciplinary practices within and outside Canada address such factors as fertility, family roles, education, careers and financial responsibilities. These models can be applied in treatment centres across the country.

## 1. Introduction

A symposium on early-age onset colorectal cancer (EAOCRC) patients organized in October 2022 addressed key issues affecting this rapidly growing population in Canada. The symposium built on the outputs of the inaugural EAOCRC conference, previously reported in this journal [1]. Readers are invited to refer to this publication for an overview of the evidence and issues in EAOCRC.

Calls to action from the previous symposium provided the basis of discussion:

- Increase awareness of the importance of EAOCRC, especially among primary care and the general public;
- Promote the earlier detection of EAOCRC in populations under age 50;
- Invest in research to optimize management pathways in EAOCRC.

### 1.1. Symposium Goals

The goals of the present symposium were to explore ways of breaking down barriers for younger Canadians, especially regarding the timely detection of colorectal cancer (CRC), and to optimize care and outcomes in EAOCRC. New evidence and leading practices were presented and discussed in a series of multidisciplinary panels tasked with providing evidence and direction to help shape healthcare policies and practices in Canada. 

### 1.2. Symposium Organization

The symposium was organized under the auspices of CCRAN, a CRC patient and caregiver support, education and advocacy network in Canada. It was co-chaired by Dr. Michael Raphael and Dr. Sharlene Gill. Facilitation was provided by Dr. Monika Slovinec D’Angelo. An Expert Steering Committee provided direction on the symposium objectives, agenda, speakers and invited participants. The Committee included Dr. Michael Raphael (Odette Cancer Centre, Sunnybrook Health Sciences Centre, Toronto, ON, Canada), Dr. Sharlene Gill (Division of Medical Oncology, University of British Columbia, Vancouver, BC, Canada), Dr. Darren Brenner (Departments of Oncology and Community Health Sciences, University of Calgary, Calgary, AB, Canada), Dr. Mary De Vera (Department of Pharmaceutical Sciences, University of British Columbia, Vancouver, BC, Canada), Dr. Petra Wildgoose (Young Adult Colorectal Cancer Program, Sunnybrook Health Sciences Centre, Toronto, ON, Canada), Dr. Clarence Wong (Division of Gastroenterology, University of Alberta, Edmonton, AB, Canada), Dr. Daniel Schiller (Department of Surgery, University of Alberta, Edmonton, AB, Canada), Dr. Mary Jane Esplen (Department of Psychiatry, University of Toronto, Toronto, ON, Canada) and Dr. Christopher Lieu (Division of Medical Oncology, University of Colorado, Boulder, CO, USA).

### 1.3. Participants

Participants included 212 health care professionals, patients and caregivers, and health care policy makers. Registrants were from Canada, the United States, South Africa, Australia, Malaysia and several European countries.

### 1.4. Agenda

The meeting agenda is presented in Table 1. All sessions were held virtually.

### 1.5. Emerging Themes

Two major themes that emerged from the symposium are the focus of this publication:

- The need for improvement in the early detection of CRC in younger populations;
- The application of leading models of care to meet the unique needs in the treatment of younger CRC patients.

## 2. Breaking Down the Barriers to the Early Detection of CRC in Younger Populations

Priorities for action identified at the symposium were to improve the early detection of CRC in younger Canadians by considering lowering the screening age to 45, and by addressing the unique needs of younger adults.

### 2.1. CRC Screening Programs in Canada

Screening for CRC has remained an important public health objective in Canada since organized programs began in 2007. CRC is the second leading cause of cancer deaths in Canada [2] and early detection has been shown to improve health outcomes [3]. Timely detection can save lives: 5-year survival rates for CRC are 90% for patients diagnosed at Stage 1, whereas at Stage 4 they are less than 15% [2].

In Canada, organized CRC screening programs are based on the Canadian Task Force on Preventive Health Care 2016 guidelines, which recommend the screening of adults aged 50 to 74 at normal risk using a fecal occult blood test (either the guaiac fecal occult blood test (gFOBT) or fecal immunochemical test (FIT)) every two years or flexible sigmoidoscopy every 10 years [4]. Unlike other countries, such as the United States, colonoscopy is not a routine screening modality but is reserved for high-risk patients and for diagnostic purposes. 

CRC-screening participation rates have been steadily increasing towards a countrywide average of 60% of the target population [5] and are expected to continue to rise to meet or exceed the goal of 70%. The rise in screening participation is believed to have contributed the steady decline in incidence of CRC seen during the past two decades [3,5]. 

### 2.2. Unique Needs of Younger Patients for Early Detection

Contrary to the declining rates of CRC observed in Canadians aged 50–74, the incidence of CRC in Canadians under 50 has been rising rapidly since 2000. The population born since 1980 is now 2 to 2.5 times more likely to be diagnosed with CRC than were previous generations at the same age [6]. In the United States, EAOCRC is the leading cause of death among age groups 20 to 39 and 40–49 [7].

Unique features of the disease in the younger age group predispose individuals to greater risk. Tumours in EAOCRC patients are diagnosed at later stages (60% are diagnosed at Stage III or IV compared with 50% in persons over age 50 [8]) and are more likely to be located in the rectum [9,10]. A recent study of Canadian patients under the age of 50 showed that mortality rates from rectal cancer increased significantly from 2000 to 2018 [11]. The regimens needed to treat these cancers have potentially detrimental impacts on younger patients and their families, and require significant healthcare system resources.

### 2.3. How Well Does the Current Crc Screening System Work for Canadians under Age 50?

The current colorectal cancer screening guidelines in Canada fail to meet the needs of patients under the age of 50. While younger patients are excluded from organized screening programs due to their age, other barriers to early detection in this age group also require consideration.

#### Gaps in the Current System

**Figure 1:**
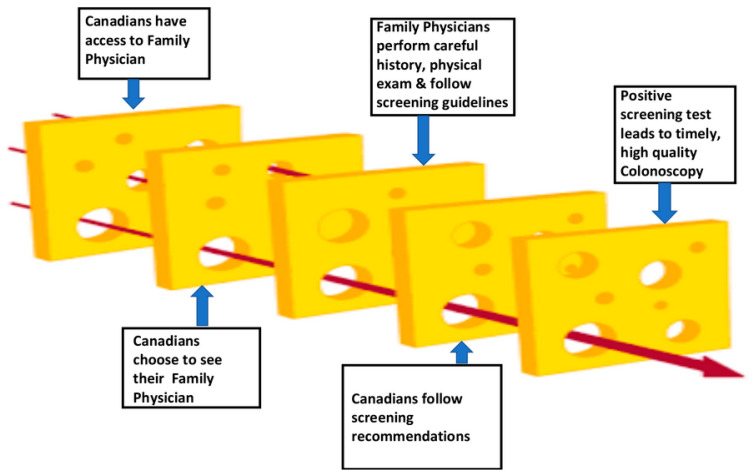
The “Swiss Cheese” model of accident causation, as applied to CRC screening. Early detection of CRC is hindered when gaps in the healthcare system align to prevent patients from receiving high quality, timely colonoscopy. Reproduced with permission from Dr. Dan Schiller, University of Alberta.

Participation in screening depends on several elements aligning at the point where a patient needs medical attention. The “Swiss cheese model of accident causation”, originally developed for the aviation industry [12], has been widely used in healthcare settings to identify the causes of medical errors. As shown in Figure 1, in the context of CRC screening, the slices of cheese represent the sequence of steps that younger Canadians would undertake. The holes in the cheese depict the gaps at each stage of the process that may delay or prevent access to timely, high-quality colonoscopy.

The first step of the process requires that Canadians have access to a family physician. The shortage of primary care providers in Canada creates a significant barrier to access to CRC screening for younger Canadians. While one in five Canadian adults does not have a regular family doctor, this figure rises to 31% of Canadians aged 18–34 [13]. For younger Canadians, this situation means that there is less continuity of care. Those with a family history of CRC may be less likely to be advised to be screened at an earlier age, and not having a primary care provider may dissuade younger patients from seeking medical attention for symptoms of CRC or for the symptoms to be recognized as unusual by the physician. Also, since younger persons of average risk are excluded from organized screening programs, this backstop of receiving a direct invitation for screening is not in place.

The second element in the diagram depicts Canadians choosing to see their primary care provider. Younger persons are less likely to seek medical attention for symptoms of CRC. In a study from the United States, delays between the onset of symptoms and treatment for rectal cancer were much longer for younger patients (217 days) compared to their counterparts over 50 (29.5 days) and were due primarily to younger patients not presenting to their primary care provider in a timely manner [14].

A lack of public education and/or misinformation regarding the importance of both a family history of CRC and of its symptoms may contribute to the delay in presentation by younger patients. A recent survey of EAOCRC patients in the United States and Canada found that most were unaware that a family history of CRC placed them at increased risk. The same study found that because symptoms of CRC (including changes in bowel habits, rectal bleeding, and symptoms associated with anemia) are non-specific and because cancer may not be suspected, many younger patients do not seek medical attention promptly [8]. A similar survey conducted in Canada found that less than half of respondents were aware of CRC risk factors, with only 30% knowing the signs of CRC before their diagnosis [15].

The third element of the model shows that primary care providers perform a careful history and physical examination and that they follow screening guidelines. Taking a family history of CRC is important for all patients, regardless of age, and especially for those under 50. Compared to Canadian adults who have no family history of CRC, Canadians younger than 50 who have a positive family history (i.e., one or more first-degree relatives: parent, brother, sister or child) carry a four-fold increased risk of developing CRC, whereas adults over 50 have a two-fold increased risk [16,17]. In Canada, individuals who have a family history of CRC are eligible for screening at age 40. A major gap in the health care system is due to many primary care providers not routinely taking a family history [18,19,20] and are, therefore, not designating these patients as being high-risk.

Another gap is that symptoms may not be taken seriously by all primary care providers. The previously mentioned survey of EAOCRC patients in Canada and the United States reported that 40% of respondents felt that their symptoms were dismissed by doctors; this finding was even more significant in the 19–39-year age range [8]. The recent Canadian survey of EAOCRC patients reported that 55% felt that their signs and symptoms of CRC were initially dismissed due to their younger age [15].

The fourth element shows that Canadians follow screening recommendations. In Canada, responsibility for following CRC screening guidelines is shared between primary care providers, provincial health authorities and patients. In Canada, CRC screening of average-risk individuals usually consists of FIT testing and colonoscopy is reserved for high-risk patients or to confirm a diagnosis. Organized CRC screening programs administer recruitment, reminder and promotional strategies to invite eligible individuals to screen as per guidelines. However, persons under age 50 are included only if they have been identified as high-risk by their primary care provider.

Adherence to CRC screening recommendations is below targets in Canada. A recent study found that adherence among Canadians aged 50 to 74 ranged from 16.6% to 47.7%, compared to the national target of ≥60% [21]. An earlier Canadian study reported low (<50%) adherence to recommended CRC screening in average and moderate risk strata [22]. An international review of published studies found that screening adherence among adults with a family history of CRC was under 50% and was even lower among those under age 50 [20]. The same study revealed that screening was facilitated by having a provider’s recommendation, multiple affected relatives and family encouragement.

The final element of the model shows that a positive screening leads to timely, high-quality colonoscopy. In Canada, access to follow-up colonoscopy does not meet the national target. The latest figures (from 2017–2018) show that the percentage of colonoscopies performed within 60 days ranged from 10.5% in Yukon to 77.1% in Newfoundland and Labrador. No jurisdiction reached the target of 90% [23].

The foregoing discussion reveals several consistent themes. First is the lack of health system capacity both in primary care and colonoscopy. Secondly, there is a lack of public and primary care education about the risks of CRC in younger patients. A focused strategy is needed about the symptoms and risk factors of CRC, the rising incidence in younger people, and the importance of family history and seeking medical attention for symptoms. For primary care providers, pathways outlining clear steps to the investigation and follow-up of CRC symptoms are currently available online [24,25,26].

While colonoscopy is a CRC screening tool in the United States, it currently is not recommended for routine screening in Canada. However, patients under 50 years of age who are symptomatic or have a family history of CRC should be screened using colonoscopy [27]. A recent development which may have relevance is the finding that sigmoidoscopy reduced CRC incidence by 31% and deaths by 50% in women and men aged 55 to 64 years [28], a finding which may be relevant to younger patients, since they have higher rates of rectal cancer compared to the population over 50 years of age.

The adage “the right test is the one that gets done” was emphasized by symposium participants. Adherence to the screening test and to screening intervals needs to be considered by both the patient and provider. The importance of this factor was recently brought to light by the NordiCC study, which compared asymptomatic populations of adults aged 55 to 64 who were either invited to undergo screening colonoscopy or who received usual care. While the study concluded that colonoscopy reduced the risk of colorectal cancer and related deaths, only 42% of people invited for a colonoscopy actually attended one. The study confirmed a higher acceptance of FIT vs. FOBT, and a higher acceptance of colonoscopy after FIT [29]. Factors such as the test location, preparation and feasibility for the patient should be considered on an individual basis. Ultimately, the choice of screening test should be based upon patient preference to ensure adherence and, therefore, early detection and treatment.

### 2.4. Support for Reducing the CRC Screening Age to 45 in Canada

Symposium participants were strongly in favour of reducing the starting screening age from 50 to 45 years of age. While a full health-technology assessment analysis will be needed to support a recommendation, the following issues and recent evidence should be considered.

First, the rising rates of EAOCRC in Canada and the broad array of impacts on patients, families and society are unique to a younger population. Due to the location and biological characteristics of tumours found in EAOCRC, and the later stage at diagnosis, both the disease itself and its treatment have profound negative impacts on patients’ health status and their ability to fulfill social and economic roles within their families and communities. A cost–benefit analysis of reducing the screening age should take into account patient-defined outcomes, such as infertility, the impact on family and caregivers, financial stress, and lifetime earning potential [8].

A second factor for consideration is the emergence of new evidence since the last Canadian guidelines were introduced in 2016. Of particular relevance is the decision of the United States in May 2021 to reduce the screening age to 45 [30], based on microsimulation modelling studies that showed that the early detection of tumours in the general population aged 45–49 avoids downstream treatment costs and saves lives [31]. Similar findings were recently reported in Canada: an economic evaluation of a microsimulation modelling study concluded that earlier screening may reduce CRC disease burden and add life years to the Canadian population at a modest cost. The authors recommended further evaluation of the resulting effects on colonoscopy capacity prior to advocating for guideline changes [32].

Symposium participants supported the development of a Canadian approach, which reflects the realities of our health care system. For example, colonoscopy is widely used as a screening tool in the United States, while its use in Canada is usually restricted to an investigation of findings from primary screening methods such as FIT or FOBT [33]. As mentioned earlier, sigmoidoscopy has recently been shown to decrease CRC incidence and mortality in the 55–64 age cohort [28]. Also, sigmoidoscopy was deemed to be a more cost-effective technique than colonoscopy in a study of a 40-year-old patient population in the United States [34]. Given the high proportion of EAOCRC tumours located in the lower bowel, this technique could be of value as a screening tool in the younger population. Finally, as discussed in the next section, the future development of a personalized approach to EAOCRC screening could identify those at risk through non-invasive (and less expensive) tests.

### 2.5. Future Advances: Promising Novel Screening Techniques

Any discussion of changes to healthcare policy must take into account promising innovative developments. Biomarkers and circulating tumour DNA (ctDNA) analyses are techniques that may potentially supplant FIT and sigmoidoscopy as first-line approaches in CRC screening [35,36]. Microbiome biomarker testing has also been proposed as a future complement to existing screening techniques.

Very little is currently known about the biology of EAOCRC tumours, and there is a major research thrust to build a body of knowledge in this area. Routine testing using a wide panel of biomarkers will not only help patients today but will help build a database of knowledge that will enable healthcare providers to tailor screening methods and predict the response to therapy in younger CRC patients, as is now possible for lung cancer [37], and is in clinical trials for melanoma patients [38].

In addition to ctDNA, panels of mRNA have also been studied with success in the early detection of colorectal adenoma. This technique offers an alternative to FIT, which is not sensitive and specific enough in colorectal adenoma detection because adenoma rarely leads to intestinal bleeding. It is also a non-invasive alternative to colonoscopy for the detection of colorectal adenomas.

Microbiome biomarker testing is another potential screening tool to detect early-stage CRC. Changes in microbial diversity and within the feces and mucosa can distinguish healthy persons from patients with CRC. Increasing evidence points to the gut microbiome as a critical mediator of adenoma formation and CRC risk. With further development, microbiome biomarker testing may provide a valuable complement to existing CRC screening tools [39].

The rapid development of these innovations has outpaced that of clinical trials used to evaluate new technologies. Ideally, biomarkers should be evaluated and approved for funding at the same time as chemotherapy regimens.

### 2.6. Advocating for Change: Lessons Learned from the U.S. Experience

The American Cancer Society succeeded in convincing the U.S. Preventive Task Force (USPSTF) to issue, in May 2021, a policy recommendation to reduce eligibility for primary preventative CRC screening to age 45 [40]. In this symposium, a panel of representatives from U.S. and Canadian patient advocacy organizations reviewed the steps taken to achieve this result. The key learnings and implications for policy change in Canada are summarized in Table 2.

## 3. Meeting the Unique Treatment Needs of Younger CRC Patients

In addition to reducing the age for CRC screening to 45, the unique needs of younger CRC patients call for a tailored approach to cancer care. Several initiatives were reviewed and discussed by participants.

The unique needs of EAOCRC patients reflect physiological and psychosocial differences between younger and older populations. Throughout their cancer journey, these factors can have profound impacts on health outcomes and the quality of life for younger Canadians and their families.

The performance of CRC screening programs is currently measured using evaluation methodologies and quality standards, such as wait times from screen-detected CRC diagnosis to the initiation of treatment, which accord with healthcare funding silos. These indicators are insufficient for younger patients because they reflect the experiences of patients already within the system [5] and do not encompass preventive measures such as education and access to healthcare services, which are more relevant in EAOCRC.

### 3.1. Different Needs of EAOCRC Patients

A diagnosis of CRC is devastating for all patients and is particularly disruptive to young adults because of their time of life and the biological characteristics of their disease.

Challenges faced by younger adults frequently involve children and spouses/partners, and have significant impacts on financial security, education, and careers. In a recent survey of EAOCRC patients, approximately three-quarters of the study population reported that they had children at the time of diagnosis [8]. The same survey reported that 66% of respondents took a leave of absence or quit a job or schooling because of their diagnosis.

Cancer management pathways are general in nature and are not optimized to meet the unique needs of younger patients. In the EAOCRC patient survey cited earlier, 37% of women and 16% of men reported that treatment left them infertile or sterile, yet only 31% of respondents said that a medical professional spoke to them about fertility preservation [8]. This survey also found that 65% of respondents suffered from sexual side effects following treatment, but only 50% were informed upfront of the possibility for sexual dysfunction; more than 25% of respondents said they would have considered alternative treatment if they had known [8]. Higher rates of depression are seen in younger CRC patients compared with their older counterparts, particularly among males. The highest risk of depression appears 1–2 years post-diagnosis, and the fear of recurrence is a major contributor that can impact daily functioning [41].

### 3.2. Leading Practices in the United States and Canada

The symposium presented and discussed examples of leading practices in EAOCRC care from the United States and Canada.

United States

Four treatment centres that offer specialized care of young adults with colorectal cancer participated in the symposium: the Young Adults with Cancer Program at Vanderbilt-Ingram Cancer Center; the Center for Young Onset Colorectal and Gastrointestinal Cancers at Memorial Sloan Kettering Cancer Center; the Young-Onset Colorectal Cancer Center at the Dana-Farber Cancer Institute; and the Center for Young Adult Colorectal Cancer at Massachusetts General Hospital Cancer Center. 

Common elements in each of the programs were:

- The recognition of the very different needs of patients under age 50;
- Multidisciplinary management and research;
- A broad scope of medical and psychosocial services (cancer treatment, mental health, sexual health and fertility, genetics, social work);
- Dedicated resources to educate and coordinate services after referral;
- Collaboration between the centre and family physicians;
- Championship of the creation and ongoing operation of the program.

**Figure 2:**
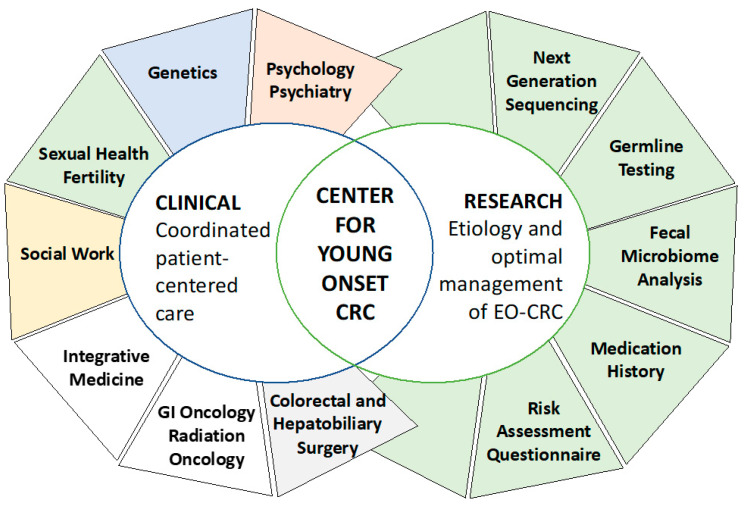
The Memorial Sloan Kettering Center for Young-Onset Colorectal Cancer’s coordinated clinical program involves gastrointestinal oncology, colorectal surgery, hepatobiliary surgery, radiation oncology, and gastroenterology, as well as support services, such as social work, fertility, sexual health, genetics, nutrition, integrative medicine, and psychology/psychiatry. In addition to these clinical services, patients are also approached for enrollment in research protocols involving tumour profiling, germline testing, and stool collection for microbiome analysis. Figure presented by Dr. Robin Mendelsohn, Co-Director, Center for Young Onset Colorectal and Gastrointestinal Cancers at Memorial Sloan Kettering Cancer Center.

The model in Figure 2 shows an array of health programs, services and integrated research programs at the Center for Young Onset Colorectal and Gastrointestinal Cancers at Memorial Sloan Kettering Cancer Center, many features of which were typical of the programs discussed.

Due to the newness of these programs, no formal evaluations have yet been conducted. Anecdotally, participants reflected that patient satisfaction is high, health services are better coordinated and more efficient, patient support beyond disease treatment are appreciated, and research studies are easier to conduct.

Canada

Sunnybrook Health Sciences Centre in Toronto, Ontario hosts the only dedicated multidisciplinary program serving EAOCRC patients in Canada. In operation since 2019, the Young Adult Colorectal Cancer Clinic is not a separate physical unit but coordinates resources across care pathways. Coordinated support by a multidisciplinary team is offered to patients throughout their journey, from diagnosis to work-up, treatment and survivorship. Comprehensive clinical care includes mental health support, medical care, genetic counselling, health system navigation, and psychosocial support for patients and their families. This approach enables holistic and continuous care, both within the institution and between the cancer centre and primary care. The establishment and continuing operation of the clinic is reliant on championship within the cancer centre.

### 3.3. Checklist of Programs and Services for EAOCRC Patients

For cancer treatment centres in Canada who serve EAOCRC patients, the following checklist of design elements may serve as a useful tool for implementing a comprehensive approach to care:

- Utilize a value-based healthcare approach to identify and prioritize the unique needs of younger patients across the continuum of care from prevention to treatment and survivorship/end-of-life.
- Implement multidisciplinary care pathways and integrated research programs
  ○ Surgical, radiation, oncology treatments;
  ○ Fertility and sexual health;
  ○ Mental health;
  ○ Genetic and biomarker testing;
  ○ Family supports (child care, financial support);
  ○ Education and collaboration with primary care and patient organizations;
  ○ Access to research studies.
- Provide dedicated resources to educate patients and staff, to coordinate and navigate services after referral, and to liaise with external partners.
- Champion the value of a specialized unit serving younger CRC patients.

## 4. Conclusions

The timely detection of CRC is crucial for people under the age of 50, given the rapidly rising rates of CRC in people under 50 in Canada (and throughout the developed world), their later stage at diagnosis, and the unique tumour characteristics of EAOCRC. Steps to achieving an earlier diagnosis include raising awareness of risk factors for EAOCRC (especially having a first-degree relative with the disease), reducing the screening age to 45. At the same time, novel approaches to screening using non-invasive tests, such as circulating tumour DNA and biomarkers, should be developed as a priority. Once diagnosed, the care of EAOCRC patients requires a broad, coordinated, multidisciplinary approach that addresses such factors as fertility, family roles, education, careers and financial responsibilities. A checklist of priorities, based on leading practices in Canada and the United States, can be applied in cancer treatment centres across the country.

## Figures and Tables

**Table 1:** Symposium agenda.

Session	Speakers
Day 1: Breaking down the barriers	
Symposium opening	Moderator: Monika Slovinec D’Angelo, Health Scientist, Conference Board of Canada Ms. Filomena Servidio-Italiano, President & CEO, CCRAN
Key learnings from CCRAN’s June 2021 Symposium	Mary A. De Vera, Epidemiologist, University of British Columbia Darren Brenner, Molecular Epidemiologist, University of Calgary
Breaking down the barriers to timely detection of colorectal cancer	Moderator: Monika Slovinec D’Angelo, Panel: Ms. Stephanie Florian, Widow of CRC patient, British Columbia Dr. Dan Schiller, Colorectal Surgical Oncologist, University of Alberta Ms. Julie Savard, Endoscopy Nurse Clinician, Jewish General Hospital, Montreal Dr. Lisa Del Giudice, Family Physician, Sunnybrook Health Sciences Centre, Toronto
Identifying and addressing the needs of younger crc patients	Moderator: Dr. Petra Wildgoose, Lead, Young Adult Colorectal Cancer Program, Sunnybrook Health Sciences Centre Panel: Mr. Marcelino Dolores, Patient Expert Dr. Mary Jane Esplen, Psychosocial Oncologist, University of Toronto Dr. Michael Raphael, GI Medical Oncologist Sunnybrook Health Sciences Center Dr. David Gurau, Reproductive Endocrinologist, Generation Fertility
Integrative therapies to address treatment-induced toxicities	Dr. Eric Marsden, Director, Marsden Centre for Excellence in Integrative Medicine
Promoting timely detection of CRC: what can we learn from the American experience?	Moderator: Dr. Robin McGee, Psychologist and Stage 4 Rectal Cancer Patient Panel: Mr. Andrew Spiegel, Executive Director, Global Colon Cancer Association (US) Ms. Martha Raymond, Executive Director, GI Cancers Alliance Inc. (US) Ms. Becky Selig, Director of Patient Education and Research, Fight Colorectal Cancer (US) Mr. Jason Gisser, Board Member, AYA Canada Ms. Dani Taylor, Manager of Programs and Partnerships, Young Adult Cancer Canada (YACC) Ms. Teresa Norris, President, HPV Global Action
Advancements in colorectal cancer treatments	Moderator: Dr. Scott Berry, GI Medical Oncologist, Kingston Health Sciences Centre Panel: Dr. Chris Lieu, GI Medical Oncologist, University of Colorado Cancer Center Dr. Kim Ma, Department of Haematology-Oncology, Jewish General Hospital, Montreal Dr. Eric Chen, GI Medical Oncologist, Princess Margaret Cancer Centre, Toronto Mr. Steve Slack, Rectal Cancer Patient
Day 2: Optimizing colorectal cancer care and outcomes	
What do we know and what have we learned from the June 2021 Symposium?	Dr. Mary A. De Vera, Epidemiologist, University of British Columbia Dr. Clarence Wong, Gastroenterologist, University of Alberta
Improving the EAOCRC patient care pathway	Moderator: Dr. Monika Slovinec D’Angelo, Health Scientist, Conference Board of Canada Panel: Ms. Hayley Painter, Young Adult mCRC Survivor Dr. Dan Schiller, Colorectal Surgical Oncologist, University of Alberta Ms. Julie Savard, Endoscopy Nurse Clinician, Jewish General Hospital, Montreal Dr. Anna Wilkinson, Family Physician, Ottawa Academic Family Health Team
Defining value and building system capacity for timely detection of EAOCRC	Moderator: Dr. Jill Tinmouth, Lead Scientist, ColonCancerCheck Program, Sunnybrook Research Institute, Toronto Panel: Dr. Petra Wildgoose, Lead, Young Adult Colorectal Cancer Program, Sunnybrook Health Sciences Centre, Toronto Dr. Yooj Ko, Medical Oncologist, Sunnybrook Health Sciences Centre, Toronto Ms. Eva Villalba, Executive Director, Quebec Cancer Coalition Mr. Jason Sutherland, Economist, Centre for Health Services and Policy Research, Vancouver Dr. Ian Bookman, Gastroenterologist, St. Joseph’s Health Centre, Hamilton Mr. Fred Horne, Horne and Associates, Edmonton
Best practices for systematically improving management of EAOCRC	Moderator: Dr. Sharlene Gill, GI Medical Oncologist, BC Cancer Agency Panel: Dr. Petra Wildgoose, Lead, Young Adult Colorectal Cancer Program, Sunnybrook Health Sciences Centre, Toronto Dr. Kimmie Ng, GI Medical Oncologist, Dana-Farber Cancer Institute, Boston Dr. Robin Mendelsohn, Gastroenterologist, Memorial Sloan Kettering Cancer Center, New York City Dr. Aparna Parikh, GI Oncologist, Massachusetts General Hospital Cancer Center, Boston Dr. Cathy Eng, GI Medical Oncologist, Vanderbilt-Ingram Cancer Centre, Nashville
Accessing innovations in CRC diagnostics and treatment	Moderator: Dr. Michael Raphael, GI Medical Oncologist, Sunnybrook Health Sciences Centre, Toronto Panel: Dr. Arvind Dasari, Medical Oncologist, MD Anderson Cancer Centre, Houston Dr. Aaron Pollett, Anatomic Pathologist, Mount Sinai Hospital, Toronto Dr. Stephanie Snow, Medical Oncologist, QEII Health Sciences Centre, Halifax Dr. Clarence Wong, Gastroenterologist, University of Alberta, Edmonton Dr. José Perea, Colorectal Surgeon, Jimenez Diaz Foundation University Hospital, Madrid, Spain Mr. Bill McGinley, Stage IV Colorectal Cancer Patient, Toronto

**Table 2:** Key lessons from U.S. advocacy experience and opportunities for Canada.

Key Lessons from U.S. Advocacy Experience	Canadian Policy Opportunities
Data are crucial to support policy change: Analyses from three microsimulation models were used to review and propose updates to existing USPSTF recommendations Measurements included health system costs and life years gained Underserved populations were also included Advocacy efforts further expanded the cost–benefit evaluation to include economic and social impacts on patients, families, and society Task forces of physicians, insurers, payers, and governments were convened to examine and agree on the data before presentation to the USPSTF	Conduct broad cost–benefit analyses: Include cost–benefit to the Canadian health system, patients, families and the economy Project short- and long-term implications Include underserved groups who are also experiencing increases in CRC (e.g., racial minorities, Indigenous populations, LGBTQ) Tailor analyses and recommendations to the realities of younger patients: FIT is too late for most symptomatic younger patients; colonoscopy is needed Randomized controlled trials are not realistic in this relatively small population Integrate the needs of younger populations into general improvements to the current system of CRC screening: Streamlined referral processes Increased prevention resources Development and promotion of national strategies through provincial guidelines. Validate analyses through multi-disciplinary expert working groups
Collaboration is essential: The American Cancer Society regularly convenes a CRC roundtable which brings together the National Cancer Institute with patient groups to look at barriers to cancer care The campaign to lower CRC screening age to 45 was supported by a broad stakeholder base including the medical community, government, patient groups, and payers	Advocate with pan-Canadian bodies and provinces Canadian Task Force on Preventive Care Provincial ministries of health Canadian Partnership Against Cancer (CPAC) could potentially convene influential organizations The Colorectal Cancer Resource and Action Network (CCRAN) is well positioned to assume a leadership role in advancing the agenda across Canada Publicize advocacy efforts with physician networks and other patient organizations Promote clinical pathways Utilize social media channels to increase public support and boost awareness

## Data Availability

Not applicable.
